# Video consent is preferred over written informed consent in pediatric rheumatology research

**DOI:** 10.1371/journal.pdig.0001067

**Published:** 2025-11-03

**Authors:** Nicholas C. Chan, Amalia R. Silberman, Megan K. Robertson, Angela R. De Castro, Marie P. Lauro, Susheen Mahmood, Tamar A. Tabrizi, Hannah Nguyen, Brian M. Feldman, Y. Ingrid Goh

**Affiliations:** 1 Division of Rheumatology, The Hospital for Sick Children, Toronto, Canada; 2 Child Health Evaluative Sciences, SickKids Research Institute, Toronto, Canada; 3 Department of Pediatrics, Department of Medicine, University of Toronto, Toronto, Canada; 4 Institute of Health Policy, Management and Evaluation, Dalla Lana School of Public Health, University of Toronto, Toronto, Canada; Shahid Beheshti University of Medical Sciences School of Dentistry, IRAN, ISLAMIC REPUBLIC OF

## Abstract

The goal of this study was to determine the difference in participant understanding, satisfaction, timing and, preference between video consent and written informed consent in a pediatric rheumatology research setting. Participants were randomized to receive either video consent or written informed consent for a registry study. After completing the first consent method, they completed a comprehension and satisfaction questionnaire. Then they received the alternate consent method and completed a second set of questionnaires. Bayesian non-parametric tests determined the difference in comprehension, satisfaction, timing and preference between video consent and written informed consent. Ninety-nine caregivers and 76 patients were randomized into video consent (n = 88) and written informed consent (n = 87) groups. Comprehension (Max = 12) and satisfaction (Max = 5) were high in both groups. There was moderate evidence supporting no difference in comprehension (median_video consent_ = 11 and median_written informed consent _= 10) and satisfaction (median_video consent_ = 4 and median_written informed consent _= 5) between video consent and written informed consent (BF_10 _= 0.225 and 0.32, respectively). The median time to complete video consent and written informed consent was 408 (95% Credible Interval (CrI): 397–412) and 360 (95% CrI: 329–391) seconds, respectively. There was decisive evidence that video consent increased the time of consent (in our sample by 48 seconds) compared to written informed consent (BF_10 _= 713). There was decisive evidence for participants preferring video consent over written informed consent (BF_10 _= 2.307x10^11^) as they thought it was easier to follow. Overall, participant understanding and satisfaction were comparable between video consent and written informed consent. Even though video consent was slightly less time efficient compared to written informed consent, video consent was highly preferred by caregivers and patients, supporting its use to obtain informed consent.

## Author summary

The most important part of enrolling someone in a research study is obtaining their informed consent. This means the person freely chooses to join the study after learning about its procedures, risks and benefits. Written informed consent is the most common way for obtaining informed consent. Usually, a person reads through a paper consent form and has a verbal discussion with a researcher. However, this method may be hard for children with varying reading abilities and comprehension skills. An alternative way is to use video consent. This method is where a person watches a video about the research study and then has a verbal discussion with a researcher. In this study video consent was compared to written informed consent for enrolling children and caregivers in a research study. Both methods had similar levels of participant understanding of the study. Also, both processes provided equally satisfying consent experience. While video consent took slightly longer, (48 seconds) compared to written informed consent, it was strongly preferred by caregivers and children. This suggests that video consent is a practical and effective alternative to written informed consent, especially when working with younger participants.

## Introduction

Informed consent is the ethical basis of enrolling human participants into research studies [1]. Written informed consent is the most common method of obtaining informed consent from research participants who have the capacity to understand. This process has potential participants read a paper consent form which is accompanied by a verbal discussion with a member of the research team [2]. After the potential participants’ questions are answered and they agree to participate, they will affirm their choice by signing a consent form [2].

Consenting children to medical research is slightly different. The age of consent for children in research studies varies by country. For example, the age of consent to medical research in the United Kingdom and New Zealand is 16 [3,4], whereas in South Africa it is 12 [5]. In Canada, there is no defined age requirement to consent children into research [6]. Specifically, Canada’s ethical guidelines for research, The Tri-Council Policy Statement: Ethical Conduct for Research Involving Humans (TCPS 2), indicates that children of any age can consent to research studies if they have the capacity to understand and appreciate the significance of the research and the implications of the risks and benefits to themselves [6,7]. As a result, it is the responsibility of the healthcare provider to assess a child’s capacity during the informed consent process. Typically, capacity is assessed by asking a child to explain the purpose, procedures, risks, and benefits of the research in their own words and acknowledge their voluntary choice to participate in research. Children who demonstrate decisional capacity can independently provide consent to participate in research and sign a written informed consent form. However, children who cannot meaningfully demonstrate decisional capacity require caregiver consent and, where possible, can assent to participate in research.

Although written informed consent is the most common practice utilized in adult and pediatric research, it has shortcomings. Specifically, the use of complex terminology (e.g., scientific jargon) on written consent forms is a significant barrier to participant understanding. A recent systematic review of 14 articles demonstrated that low comprehension about fundamental aspects of research studies (e.g., randomization, risks, and side effects) were commonly observed due to low adult health literacy [8]. Since children’s reading comprehension level and health literacy are generally lower than adults’, reading written informed consent forms may pose a greater comprehension challenge for children [9]. In cases where written informed consent was presented to children (12–17 years) with age-appropriate language, only 56% of participants were able to understand the study [10]. The variability in a child’s capacity to synthesize and process written information poses additional comprehension challenges for written informed consent in a pediatric setting [11].

Due to the shortcomings associated with written informed consent, alternative methods are needed in both adult and child populations. One potential alternative to written informed consent is the use of video consent. In this method, a pre-recorded audio supported by an animated/live action video explains the research study to potential research participants; thereafter, questions are answered by a member of the research team. Given that many individuals have varying learning styles, presenting information in a video format may appeal to visual, pictorial, auditory and verbal learners better than conventional written consent [12].

The few studies evaluating video consent, in both adults and children, have suggested multiple benefits. In adults, video consent has been shown to improve comprehension, satisfaction and to increase patient-physician discussion [13–17]. This may be especially true among groups with low levels of education and literacy [13,18,19]. Video consent has been studied for pediatric, procedure-based, clinical consent – including surgery and cancer treatments [13,14,17]. Video consent methods were significantly preferred by children compared to written informed consent, improved children’s comprehension of research studies, and facilitated more patient to physician discussion about the benefits and risks of research studies [17,20,21].

However, the effects of video consent on participant comprehension and satisfaction are highly variable between different studies. For example, when consenting children for inguinal hernia repair, video consent increased caregiver comprehension, but had no impact on satisfaction [22]. Conversely, when consenting adult patients into an oncology randomized control trial, video consent improved participant satisfaction, but had no effect on comprehension [17].

Also, few studies have assessed the effect of video consent on the duration of the consenting process, and there is currently no consensus. Two studies have concluded that video consent decreases the time to consent adult participants for surgery by up to four minutes [15,23]. However, a study by Simon, *et al.*, concluded that video consent increases the time to consent adults into a biobanking study by an average of six minutes [24].

To our knowledge, there has been no study of the impact of video consent in pediatric rheumatology research. Our study aimed to examine the effects of a video consent compared to written informed consent. Specifically, we wanted to determine if there was a difference in comprehension, satisfaction, and administrative time between video and written informed consent in pediatric rheumatology patients and their caregivers. Furthermore, we wanted to determine which format patients, and their caregivers preferred.

## Methods

### Ethical considerations

This study was approved by The Hospital for Sick Children (SickKids) Research Ethics Board (REB#:1000053563). Written informed consent was obtained from all participants and/or their caregiver, as applicable. Capacity to consent and assent was determined by the treating physician. All data collected in the study were de-identified.

The Pediatric Rheumatology Care and Outcome Improvement Network (PR-COIN) registry was used as the exemplar research study for our project [25]. Briefly, the PR-COIN registry aims to collect data from clinical notes, medications and test results of people who are diagnosed with juvenile idiopathic arthritis (JIA) in order to create a repository of information to perform research and quality improvement activities. Participants in this study were not enrolled in the PR-COIN registry as a majority of the participants were ineligible for the study, while a small minority of participants had already been enrolled.

### Development of a consent video

The research team drafted the initial script and screen play for a video explaining the PR-COIN registry. Briefly, the video outlined the following topics: the purpose and information collected by the registry, the eligibility criteria of the registry, the risk and benefits of joining the registry, the process of enrolling, withdrawing or declining to participate in the registry, and the methods to receive results generated from the registry. Feedback on the video’s face and content validity was sought from a convenience sample including multiple involved parties including patients, caregivers, rheumatology clinical and research team members, and members of both the ethics and quality improvement office. Multiple revisions occurred in response to the feedback received, such as order of information presented, the content of the video as well as the vocabulary used in the audio. The video was developed using a whiteboard animation style using Video Scribe 3.7.3622 and Adobe Illustrator 2020, with audio recorded using QuickTime Player 10.4. The video was reviewed by the same involved parties listed above and was iteratively revised until no additional suggestions were received. The final video was 6 minutes, and 48 seconds long could be placed on a tablet or desktop to study participants (S1 Video).

### Written informed consent form

The written informed consent form used for this study was a version of the PR-COIN registry consent form that had been previously approved by the SickKids Research Ethics Board. Briefly, the written informed consent form outlined the following topics: the purpose and information collected by the registry, eligibility criteria of the study, the risk and benefits of joining the registry, the process of enrolling, withdrawing or declining to participate in the registry, and the methods to receive results generated from the registry (S1 Text).

### Study procedure

**Fig 1:**
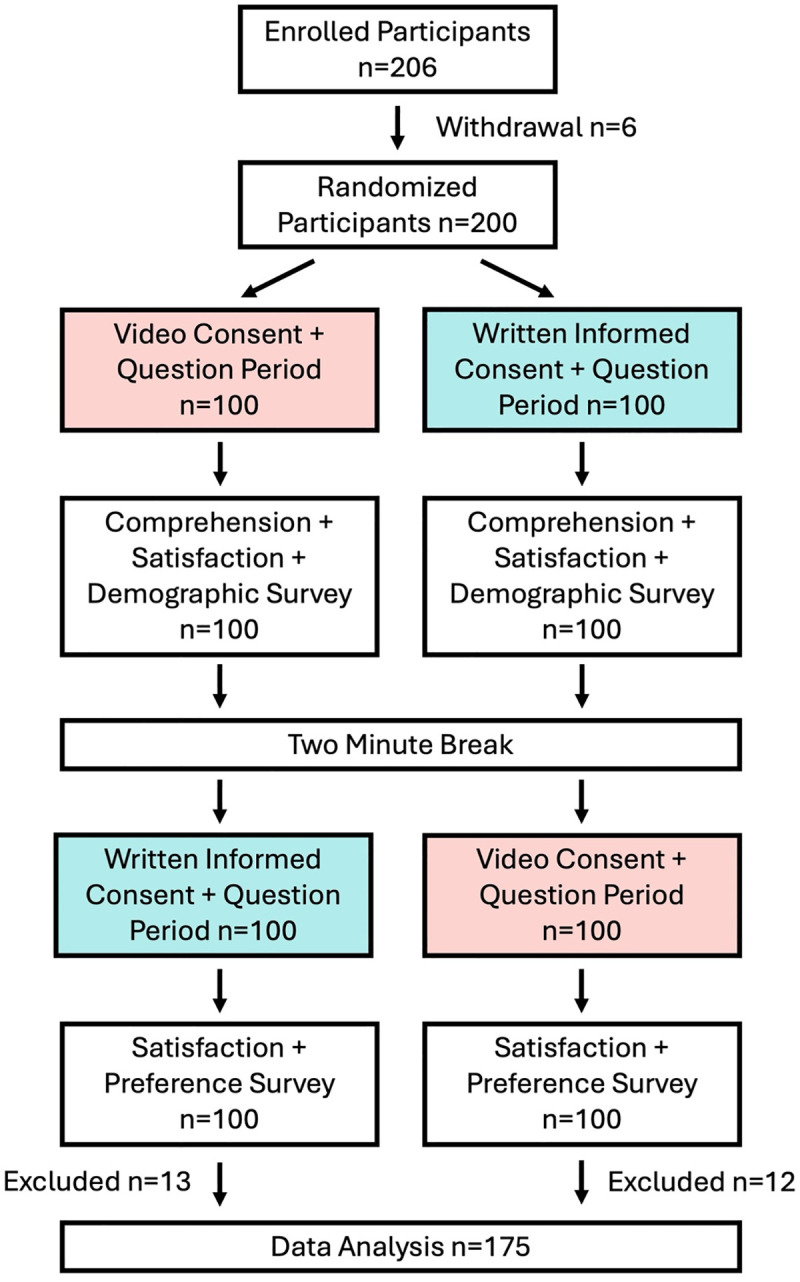
Consort Flow Diagram.

Patients (10–18 years old) and caregivers were stratified by language proficiency (i.e., English as a first language or English as a second language) and role (i.e., patient or caregiver). Then, the order of receiving consent information (either video or written format) was randomized (random.org). Participants were instructed by a member of the research team to read the written informed consent form or watch the consent video at their own pace (i.e., participants could freely reread the consent form or rewind the video if needed). After the research team member finished their instructions, they started a timer in REDCap Version 12.3.3 and distanced themselves from the participant to review the materials independently. Upon completing the first consent format, the participant verbally told the research team member that they had finished. Then, the research team member asked the participant if they had questions and answered them accordingly. Once all the participant’s questions were answered, the research team member stopped the timer on REDCap. Next, the participants completed a comprehension and satisfaction questionnaire, as well as a demographic survey on REDCap. After a two-minute break, participants were then presented the same information using the other consent format following the same procedure as above. After the timer was stopped the participant completed a second satisfaction and preference questionnaire (Fig 1).

### Randomization

The order of consent (video first or written informed first) was determined by a 4-6-8 randomization module using random.org. This method ensured equal ordering for the 4 study groups (1. Caregivers whose first language was English, 2. Caregivers whose first language was not English, 3. Patients whose first language was English, and 4. Patients whose first language was not English).

### Demographic survey

The demographic survey collected participants’ race/ethnic origin according to the Statistics Canada Classification (Version September 18, 2017), biological sex, experience with research, and highest level of completed education (S2 Text).

### Comprehension questionnaire

The comprehension questionnaire consisted of 12 true or false questions that assessed important study design elements (i.e., purpose, study protocol), risks (i.e., data breach) and eligibility criteria (S3 Text). Our questionnaire was administered on a tablet through REDCap and was modeled on one used by Mack *et al.* [26]. The survey comprised of 11 factual questions and 1 inference question (“are you eligible to participate in the study?”).

### Satisfaction questionnaire

Satisfaction, defined as the overall enjoyment, ease of use and fulfillment of one’s expectation for the overall informed consent process, was measured using a 5-point Likert scale (strongly agree – strongly disagree) to the following question: “I was satisfied with the way the consent was performed”. The satisfaction questionnaire was administered on a tablet through REDCap and the satisfaction Likert scale choices were converted into an ordinal scale, where strongly disagree = 1 and strongly agree = 5 (S4 Text).

### Preference questionnaire

Preference, defined as the participant’s favored method of consent delivery, was measured after the participant completed both consent methods. We asked, “When comparing both types of consent my preference is:”, participants had the option selecting video consent, written consent, or no preference on a tablet through REDCap. Participants were asked to justify their selection using open text (S5 Text).

### Validation of comprehension, satisfaction and preference questionnaire

The bespoke comprehension, satisfaction and preference questionnaire were specifically designed for this study. Content validity of the comprehension questionnaire was measured with a 7-point Likert scale to the following question “Do you think this questionnaire covers all the relevant topics discussed in the PR-COIN research consent form?”. Face validity of the comprehension, satisfaction and preference questionnaires were assessed with 7-point Likert scale to the following questions: 1) “Do you think this questionnaire accurately measures a person’s comprehension of the PR-COIN research consent form?” 2) “Do you think this questionnaire accurately measures a person’s satisfaction after an informed consent experience?” 3) “Do you think this questionnaire accurately measures which consent process a person prefers?” (S6 Text). A sample of research students/volunteers, researchers, physicians, research coordinators, and principal investigators who were not involved in the research study answered the 7-point Likert scales. Likert scale choices were converted to an interval scale where strongly disagree = 1 and strongly agree = 7.

### Timing

The administration time for each consent method was recorded on a tablet through REDCap. The consent administration time included the time to review the video or written consent form at the participant’s own pace, and the time to answer any additional questions arising from the video or written consent form.

### Sampling

Participants were consecutively recruited from the SickKids rheumatology clinic from June 6, 2022 to October 20, 2022. Any patient and/or their accompanying caregiver who had a scheduled appointment at the rheumatology clinic were eligible to participate in the study, irrespective of their diagnosis or disease severity. There were no exclusion criteria.

### Sample size

We used 5,000 Markov chain Monte Carlo simulations with a Cauchy prior of scale 0.707 on TensorFlow version 2.18 to determine that a sample size of 87 participants per group would be sufficient to discern a 10% difference in comprehension, satisfaction, and preference between the two study groups with tight credible intervals which excluded 0 (95% Credible Interval (CrI): 0.05-0.17). We aimed to enroll 206 participants to account for a 15% dropout rate.

### Statistical analysis

All statistical tests were performed using the R Statistical Software (Version 4.1.2) and JASP (Version 0.16.3) [27, 28]. Each participant received both methods of consent. Comprehension was assessed only after the first method was applied; differences between the methods was tested using a Bayesian Mann-Whitney U test. A Cauchy prior with a scale of 0.707 was used for all Bayesian tests. Satisfaction and timing were assessed with a Bayesian Wilcoxon paired sign test. Preference for either method was tested using a Bayesian Fisher’s exact test.

## Results

We recruited 206 participants from SickKids’ rheumatology clinic. Six participants withdrew from the study (no specified reason) and 25 participants were removed by investigators due to interruptions in the data collection or incomplete data. A total of 175 participants were included in the final analyzed sample.

### Demographics

Ninety-nine caregivers (median age range 45–49 years) and 76 patients (median age range 10–14 years) participated (Table 1). Most of our participants spoke English as their first language (n = 121). The most represented race/ethnic origin in our sample were Asian participants. The most common diagnosis amongst the patients in our sample was JIA followed by childhood-onset systemic lupus erythematosus (Table 2). Lastly, there was a higher proportion of female patients and caregivers in our sample. This sex disparity was expected as a there is a female sex bias for most pediatric rheumatic diseases and patients often accompanied their mother during their clinic visit [29].

**Table 1:** Demographics of Study Participants.

Characteristics	Video Consent First Group (n = 88)	Written Informed Consent First Group (n = 87)
Sex		
Male	25	32
Female	62	55
Prefer not to answer	1	0
Participant role		
Patient	37	39
Caregiver	51	48
Language Proficiency		
Participants first language Was English	61	60
Participants first language was not English	27	27
Age in Years		
10-14	17	22
15-19	20	17
20-24	0	1
25-29	1	1
30-34	2	1
35-39	9	4
40-44	12	12
45-49	16	18
50-54	9	10
55+	2	1
Race/Ethnic Origin		
Asian	28	36
European	27	16
North American	17	13
South American	8	9
African	3	6
Caribbean	4	5
Indigenous peoples of Canada	2	1
No Response	13	6

**Table 2:** Distribution of Patient Diagnosis.

Diagnosis	Patients (n = 76)
Juvenile Idiopathic Arthritis (JIA)	20
Childhood-onset Systemic Lupus Erythematosus (cSLE)	16
Not Yet Diagnosed	16
Juvenile Dermatomyositis	5
Chronic Nonbacterial Osteomyelitis	6
Uveitis	3
Vasculitis	4
Scleroderma	2
Autoinflammatory	1
Kikuchi Disease	1
Chronic Pain	1
Kawasaki Disease	1

Thirty participants validated our comprehension, satisfaction and preference questionnaires (Table 3). The majority of the validation cohort completed a graduate level degree and spent around 50–70% of their workday performing research. Half of the validation cohort were research students/volunteers, while the rest of the validation cohort consisted of research coordinators, physicians, principal investigators and researchers.

**Table 3:** Demographic of the Validation Cohort.

Characteristic	Cohort (n = 30)
Participant Education	
Some Undergraduate Studies	5
Completed Undergraduate Degree	4
Some Graduate Studies	5
Completed Graduate Degree	16
Exposure to Research: Approximate percentage (%) of participant’s workday	
<10	1
30	8
50	8
70	5
90	5
100	3
Participant Role	
Research Student/Volunteer	15
Researcher	4
Research Coordinator	3
Physician	4
Principal Investigator	4

### Comprehension

65% (95% CrI: 52.3%-75.4%) of the validation cohort “*Agreed or Strongly Agreed*” that the comprehension questionnaire adequately covered all relevant topics of the PR-COIN research study (content validity). 80% (95% CrI: 62.5%-90.4%) of the validation cohort “*Agreed or Strongly Agreed*” that the comprehension questionnaire sufficiently assessed a participant’s comprehension of the PR-COIN research study (face validity).

**Fig 2:**
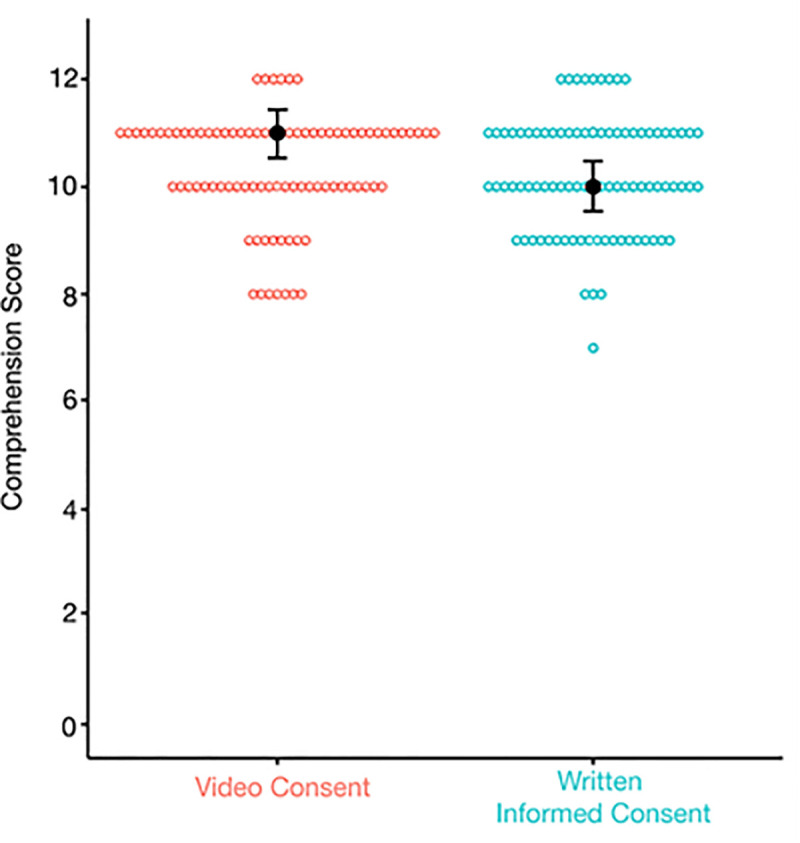
Difference in Median Comprehension Score Between Video Consent and Written Informed Consent. Data represented as median ± credible interval (CrI) and dot plot of comprehension scores (out of 12) for participants who received video consent (n = 88) compared to those who received written informed consent (n = 87). Difference in median comprehension score was determined with Bayesian Mann-Whitney U test, BF10 = 0.225.

The median comprehension score (out of 12 points) for the video consent group was 11 (95% Credible Interval (CrI): 10.68–11.22). The median comprehension score for the written informed consent group was 10 (95% CrI: 9.77–10.70). There was moderate evidence for no difference in comprehension scores between the video consent and written informed consent group (BF_10 _= 0.225) (Fig 2) [30]. When the data was stratified by the types of questions, factual questions (Q1-Q11) or inferential question (Q12), there was moderate evidence for no difference in comprehension score between video consent and written informed consent (BF_10 _= 0.277 and 0.19, respectively) [30]. The percent correctness of factual questions for the video and written informed consent groups were 90.8% (95% CrI: 89.1%–92.3%) and 89.3% (95% CrI: 87.5%–91.2%), respectively. The percent correctness for the inferential question for the video and written informed consent groups were 35.2% (95% CrI: 25%–45.4%) and 35.6% (95% CrI: 25.4%–45.9%), respectively. Comprehension scores were also assessed when our sample was stratified by education level. There was anecdotal evidence for no difference in comprehension score between caregivers with high school, undergraduate and master or higher education (BF_10 _= 0.418) [30]. Similarly, there was anecdotal evidence for no difference in comprehension score between patients with middle school and high school education (BF_10 _= 0.514) [30]. Finally, subgroup analysis restricted by language proficiency (i.e., only participants with English as a first language), participant role (i.e., only patients, or only caregivers) and patient diagnosis (i.e., JIA vs non-JIA patients, given the PR-COIN study was recruiting JIA patients) was done for all the analyses above and found similar results. Taken together, there was no difference in comprehension score between video and written consent group, when accounting for the type of questions, education level, language proficiency, participant role and patient diagnosis.

### Satisfaction

81% (95% CrI: 66.2%-92.5%) of the validation cohort “*Agreed or Strongly Agreed*” that the satisfaction questionnaire adequately measured a participant’s satisfaction of their consent experience (face validity).

**Fig 3:**
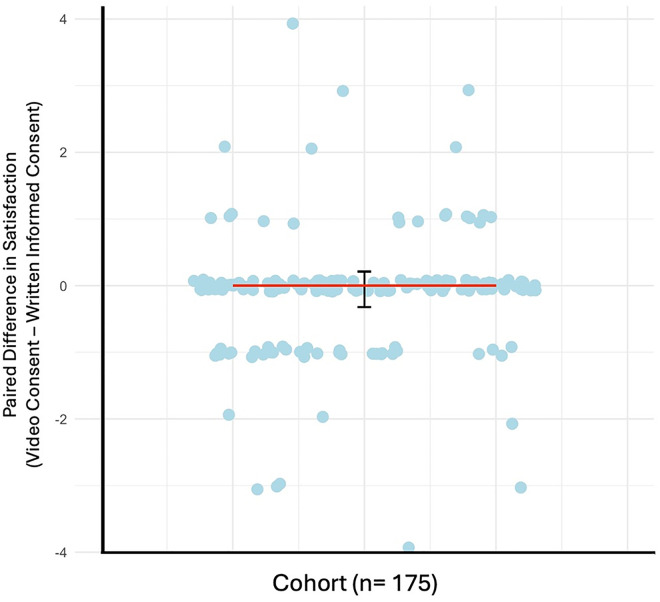
Paired Difference in Median Satisfaction Scores (Video Consent – Written Informed Consent). Data represented as median ± credible interval (CrI) and dot plot of the paired difference of the satisfaction score for video consent subtracted by the satisfaction score for written informed consent for each participant in our cohort (n = 175). Paired difference in satisfaction scores was determined with a Paired Bayesian Mann-Whitney U test, BF10 = 0.32.

The median satisfaction score for video consent was 4, where 87.4% of participants scored their satisfaction a 4 or higher. The median satisfaction score for written informed consent was 5, where 90.3% of participants scored their satisfaction a 4 or higher. The median paired difference was 0 (95% CrI: -0.32–0.21) and there was moderate evidence for no difference in satisfaction (BF_10 _= 0.32, Fig 3) [30]. Additional subgroup analysis restricted by language proficiency and participant role and found similar results. Taken together, there was moderate evidence for no difference in satisfaction scores between video consent and written informed consent accounting for language proficiency and participant role.

Regarding the ordering of consent formats, there was moderate evidence for no difference in the median satisfaction scores (BF_10 _= 0.31) between participants who experience video consent first (median = 4, 95% CrI: 3.55–4.45) compared to video consent second (median = 5, 95% CrI: 4.39–5.61). Similarly, there was moderate evidence for no difference in the median satisfaction scores (BF_10 _= 0.2) between participants who experienced written informed consent first (median = 4, 95% CrI: 3.98–4.39) compared to written informed consent second (median = 4, 95% CrI: 3.80–4.32). Collectively, the order of the consent formats does not affect the satisfaction scores given to the video or written informed consent process.

### Timing

**Fig 4:**
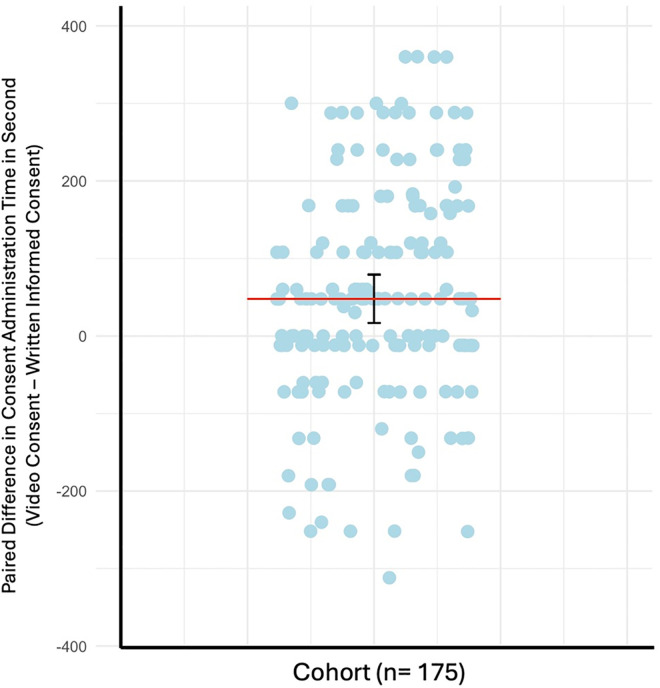
Paired Difference in Median Time to Complete Consent in seconds (Video Consent – Written Informed Consent). Data represented as median ± credible interval (CrI) and dot plot of the paired difference in the time to complete video consent subtracted by the time to complete written informed consent for each participant in our cohort (n = 175). Paired difference in time to complete the consent process was determined with a Paired Bayesian Mann-Whitney U test, BF10 = 713.

The median total time to complete the video consent was 408 seconds (95% CrI: 397–412 seconds) with a range of 120–600 seconds. Conversely, the median time to complete written informed consent process was 360 seconds (95% CrI: 329–391 seconds) with a range of 60–720 seconds. The median paired difference of time to complete the consent process (time to complete video consent – time to complete written informed consent) was 48 seconds (95% CrI: 17–79 seconds). There was decisive evidence (BF_10 _= 713) that the total time to complete video consent was longer than written informed consent (Fig 4) [30]. Subgroup analyses were performed restricting our sample by language proficiency and participant role. Similar results were observed when we stratified the cohort by caregivers and participants who first language was English (BF_10 _= 331 and BF_10 _= 140, respectively). However, when the cohort was stratified to only include patients, the median time to complete video consent was 408 seconds (95% CrI: 383–433) with a range of 120–600 seconds, and the median time to complete written informed consent was 360 seconds (95% CrI: 228–492) with a range of 60–720 seconds. Even though median paired difference of time to complete the consent process remained at 48 seconds (95% CrI: -93–189), the strength of the evidence weakened from decisive, to anecdotal evidence (BF_10 _= 1.5) that the time to complete video consent was longer than written informed consent in this subgroup. Similarly, when the cohort was stratified by participants who first language was not English, the median time to complete video consent was 408 seconds (95% CrI: 396–420) with a range of 120–600 seconds and the median time to complete written informed consent was 360 seconds (95% CrI: 180–540) with a range of 120–660 seconds. Even though median paired difference of time to complete the consent process remained at 48 (95% CrI: -96–169), the strength of the evidence weakened from decisive to moderate evidence (BF_10 _= 5.9) that the time to complete video consent was longer than written informed consent in this subgroup. Taken together, there was decisive evidence that the time to complete video consent was longer compared to written informed consent for caregivers and participants with English as their first language. However, the strength of evidence that video consent was longer than written informed consent was weaker for patients and participants with a first language that is not English.

Regarding the ordering of the consent formats, there was moderate evidence for no difference in the median time to complete video consent (BF_10 _= 0.19) between participants who experience video consent first (median = 408, 95% CrI: 400–409) compared to participants who experience video consent second (median = 408, 95% CrI: 390–410). Similarly, there was substantial evidence for no difference in the median time to complete written informed consent (BF_10 _= 0.18) between participants who experience this format first (median = 360, 95% CrI: 320–376) compared to participants who experience this format second (median = 360, 95% CrI: 324–386). Collectively, the order of the consent formats does not affect the time to complete video or written informed consent.

### Preference

84% (95% CrI: 70.2%-94.5%) of the validation cohort “*Agreed or Strongly Agreed*” that the preference questionnaire adequately measured a participant’s most preferred consent experience (face validity).

**Fig 5:**
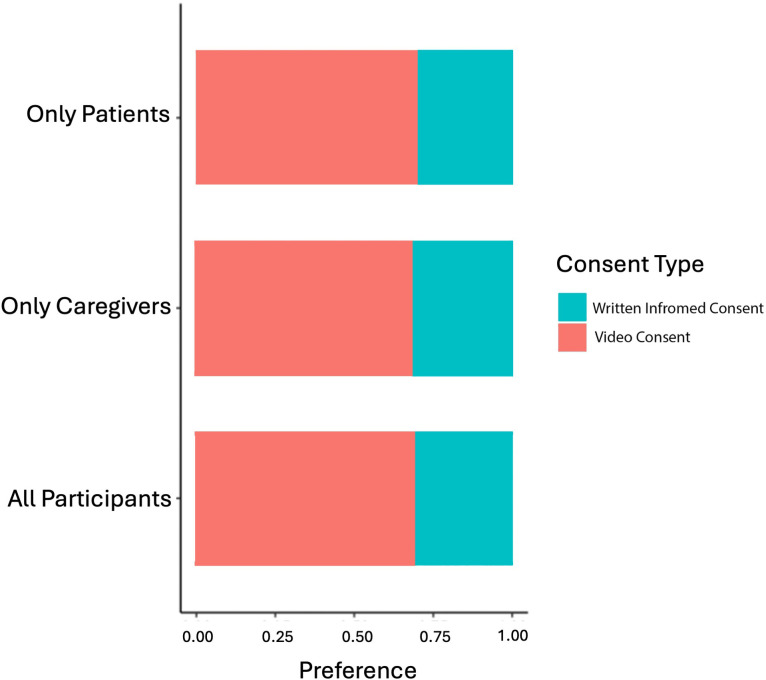
Proportions of Participants Who Preferred Video and Written Informed Consent. (npatients = 76, ncaregivers = 99, ntota l = 175). Difference in the proportion of participants who preferred video and written informed consent was determined with Bayesian Fisher’s exact test, BF10 = 2.307x10^11^.

There was decisive evidence for video consent being preferred over written informed consent (BF_10 _= 2.307 x 10^11^) [30]. 69.07% of participants preferred video consent over written informed consent (Fig 5). Additional subgroup analysis, restricted by language and participant role and found similar results.

Regarding the ordering of consent formats, there was decisive evidence that a greater proportion of participants preferred video consent when it was presented as the second consent format compared to the first format (BF_10 _= 35.7). When video consent was presented as the second consent format (or written informed consent was the first format), 85% (n = 74 of 87) of participants preferred video consent over written informed consent. Conversely, when video consent was presented as the first consent format (or written informed consent was the second format), 62% (n = 54 of 88) of participants preferred video consent over written informed consent. Taken together, video consent was preferred over written informed consent regardless of the order of consent format. Nevertheless, when video consent was presented as the second consent format, a greater proportion of participants preferred it over written informed consent compared to when video consent was presented as the first format. Thus, the order of consent formats, to a degree, moderated participants preference.

The most common reasons that participants preferred video consent over written informed consent were: 1) the video was easier to understand than the paper consent form; 2) the video was easier to follow and kept their attention; 3) the video better fit to their learning style; and 4) the images were very helpful for children. The most common reasons that participants preferred written informed consent over video consent: 1) the paper form gave the chance to review at one’s own pace and flip back to previous sections; 2) the paper form provided more details than the consent video; and 3) the paper form had fewer distracting visuals.

## Discussion

Our results suggest that participant comprehension and satisfaction are comparable between video and written informed consent. Although, video consent slightly increases the time to complete consent compared to written informed consent, if given the option, participants *prefer* video consent over written informed consent.

### Comprehension

Our result of no difference in comprehension rates between video and written informed consent conflicts with the published literature, as a majority of studies with similar randomized and interventional designs in both pediatric and adult populations concluded that video consent improved participant comprehension [1,21,22,31–33]. Furthermore, we did not observe any difference in comprehension score when our sample was stratified by education level, which conflicts with the published literature (education level and comprehension have previously been shown to be strongly positively associated) [34,35]. However, our results are not alarming as both consent methods achieved high comprehension scores, specifically for factual questions, suggesting that unlike the studies in the literature both methods effectively informed participants. This may be attributed to the fact that we were very mindful of word choice when developing both our video and written consent form as we recognized the potential comprehension barriers of our population. The discrepancy in correctness for the inferential question (“are you eligible for participate in this study”) may be due to the inherent difficulty of inferential questions compared to factual questions [36]. Alternatively, there could have been a lack of understanding around participant eligibility in both consent mediums. Thus, additional research comparing the difference in participant understanding of inferential and factual questions are needed.

### Satisfaction

We saw no difference in satisfaction rates between the video and written informed consent groups, and this is consistent with the current literature [1,21,22,31–33]. Nevertheless, our highly left-skewed distribution of satisfaction rating for our video and written informed consent is unusual. High satisfaction may be attributable to the high comprehension of both consent methods, but additional research is needed to determine the most important factors that lead to a highly satisfying consent experience. In addition, we believe that participant satisfaction likely differs depending on the quality, length and complexity of the consent video or the written consent form. As such, researchers must seek feedback from all involved parties to effectively design satisfying consent videos and written informed consent forms.

### Timing

Currently in the literature, there is no consensus on the time effectiveness of video consent compared to written informed consent. For example, the results of three interventional studies with a similar design to our current study had differing conclusions on the time effectiveness of video consent compared to written informed consent. Firstly, a randomized trial of 200 adults (mean age of 47), concluded that video consent increased the mean consent time for a biobank by 5.6 minutes compared to written informed consent explained verbally [15,24]. Secondly, a randomized trial of 435 adolescents and young adults (aged 13–24, mean age of 18.7) demonstrated that video consent decreased the time to consent into a vaping cessation study by 36 seconds compared to written informed consent [37]. Lastly, a randomized trial of 77 adults (median age 45) demonstrated that the time to complete video consent for rhinologic surgery did not significantly differ between the written informed consent group [38]. Currently, our results align with the by Simon *et al.* study. Consequently, our results support the conclusion that video consent is less time-efficient than written informed consent; however, the magnitude of additional time required for consent was much smaller in our study compared to the Simon *et al.*(an increase of 48 seconds compared to 5.6 minutes). Nevertheless, given the variability that exists around the time efficiency of video consent, even in similarly designed interventional studies, this issue is certainly not resolved. Thus, additional studies focusing on the time effectiveness of video consent compared to written informed consent are needed.

### Preference

Participants’ preference for video consent is consistent with results in the literature. Two independent studies found that children preferred animated consent videos over paper consent forms as they better held their attention and improved their understanding [17,20]. Additionally, it has also been shown that caregivers prefer receiving information by video consent as it better maintains their attention and caters to their diverse learning styles [39,40].

The moderating effect of the order of consent formats on participants’ preference can be explained by the recency effect. Briefly, the recency effect suggests that a participant is more likely to remember the last event compared to earlier events. Thus, when rating a specific experience, a participant will attribute more of their rating to the last event compared to earlier events, which may have faded out of recall. In practice, the recency effect has been shown to influence participants’ rating of sequential events. According to a study of 79 adults who rated the overall pleasantness of six sequential short stories, participants were more likely to derive their rating from their experience of last story compared to the first five stories (d = 0.21, p < 0.05) [41]. Consequently, given the recency effect, it is logical to observe a higher preference for video consent when it was presented as the second format because it represented the last experience prior to participants indicating their preference. Similarly, when video consent was presented first, it follows that a greater percentage of participants would indicate written informed consent as their preferred format since written informed consent was experienced last. Thus, the moderating effect that we observed from the order of consent formats on participants’ preferences is explained by the recency effect. Nevertheless, since video consent was still preferred over written informed consent irrespective of the order of the consent formats, we are confident that video consent was the most preferred consent format in our cohort.

### Ethical and moral considerations of video consent

The primary purpose of informed consent is to ensure that a potential participant understands the nature of the research, including its risks and benefits, and appreciates their right to voluntarily participate or withdraw from a study. Accordingly, researchers/physicians have a moral obligation to transparently communicate all relevant aspects of a research study, enabling participants to make an informed and autonomous decision about their involvement in research. Consequently, the human-to-human interaction and deliberation between a researcher/physician and a participant is a key ethical component of the informed consent process, particularly when decision are sensitive or complex.

Video consent, followed by a live question-and-answer period with a researcher/physician (the format used in this study), can preserve the ethical obligation for empathetic interpersonal connection while improving the consistency and addressing power imbalance issues in the informed consent process. Given the informational asymmetry that exists between researchers/physicians and participants, a power imbalance remains between the two parties [42]. Thus, a researcher/physician may exert considerable influence over a medical decision by framing the information of a research study to compel a less knowledgeable, and often stressed, participant to consent into research [43,44]. Thus, by utilizing a consent video, that has been reviewed and approved by an institution’s research ethics board, all relevant aspects of a research study should be presented in a transparent, accessible and identical manner to all participants. Consequently, video consent ensures a more standardized and equitable delivery of information to all participants, while minimizing researcher/physician-biased framing of study information. Furthermore, upon completing the consent video, newly informed participants proceed to a question-and-answer period with a research/physician. By reducing informational asymmetry between the two parties, this approach promotes a more empowering and balanced atmosphere that helps minimize the power imbalances between researchers/physicians and participants [45]. As a result, the question period provides an important opportunity for participants to seek clarification and/or voice their personal concerns. Moreover, researchers/physicians can also leverage this face-to-face interaction to confirm participants’ comprehension of the research, develop an understanding of the participants’ personal context and respond empathetically through a balanced dialogue. Ultimately, the face-to-face question period preserves the moral obligation of researchers/physicians to facilitate an empathetic deliberation with participants. In doing so, researchers/physicians can ensure that participants have enough information to make an informed and autonomous decision about their involvement in research. Collectively, video consent followed by a live question period is morally justified and improves upon the consistency and addresses the power imbalance issues that currently exist in the informed consent process.

### Implications with artificial intelligence

With the emergence of commercialized artificial intelligence tools, the ability to translate videos, auto-generate video captions and generate edited videos has become more feasible and accessible to the general public. For example, tools such as Rask AI and Dubverse.ai offer a timely (within 5–30 minutes) and cost-effective (starting at $60 for 25-minute video translation) solution to translate and generate captions to any video for over 120 languages [46,47]. According to a pediatric review of 84 studies, Hispanic and Asian/Pacific Islander non-English speaking caregivers were more likely to participate if study materials were translated in their preferred language [48]. Thus, by translating and captioning consent videos in other languages it may be possible to increase the enrolment of the underrepresented non-English speaking population in research studies [49]. Nevertheless, additional research is needed to evaluate the accuracy, time and cost effectiveness of artificial intelligence-based video translation and captioning for research informed consent materials.

Similarly, artificial intelligence video generators such as Steve AI and Pictory offer an inexpensive (starting from $15 and 19 dollars a month, respectively) and time effective (within 1–10 minutes) solution to generate animated videos with an accompanying voice over from a text script [50,51]. Thus, artificial intelligence video generators have made it possible for researchers with limited technical skills in video editing, animation or audio recording to create professionally animated consent videos in minutes. Consequently, these tools have minimized the technical barriers of creating consent videos, making the adoption of video consent more feasible in research settings.

### Limitations

Our conclusions must be considered in the light of a few potential limitations. First, there were a small number of male participants in our sample. This disparity was expected as we enrolled a high number of individuals with a diagnosis of systemic lupus erythematosus and mothers more often accompanied their children to clinic and were available to enroll. Nevertheless, when our sample was stratified by sex for subgroup analysis our results were similar [26].

Second, our study had a much smaller number of participants who did not speak English as a first language. Given that the primary language that is used and taught in public schools in Toronto is English, this language proficiency distribution is expected. In addition, all caregivers and children who identified as speaking English as a second language, self-reported that they were able to hold a conversation and read a news article in English for at least five years. Our study may have excluded potential participants with extremely limited English capabilities as participants needed to consent in English in order to participate. Additional research with a focus on participants with extremely limited English (or whichever language consent is being presented in) is still needed.

Thirdly, the results of our study are, perhaps, generalizable only for acquiring consent for registry-based research studies. Consequently, video consent provides comparable levels of participant comprehension and satisfaction to written informed consent, while being the preferred format for registry enrollment. Nevertheless, the benefits of video consent over written inform consent may vary depending on the specific research study design. For example, when consenting pediatric leukemia patients into a Phase III randomized control trial, video consent improved participant satisfaction, but had no effect on comprehension [17]. Thus, additional research is needed to access whether video consent is a suitable alternative to written informed consent for different types of study designs (i.e., observational, interventional or longitudinal cohort studies).

### Conclusion

Our study demonstrated that video consent ensured high participant comprehension and satisfaction which was comparable to written informed consent. Although video consent did not reduce the total time to complete the consent process, it offers a more standardized experience that may mitigate the power imbalances that exist in the consenting process and was substantially more preferred by patients and caregivers compared to written informed consent. Given the rapidly evolving trajectory of artificial intelligence, it is likely that auto-generated captions, and full video language translation will continue to improve, thereby increasing the accessibility of video consent and improving the representation of non-English speakers in research. In conclusion, our findings suggest that video consent is a suitable alternative to written informed consent.

## Supporting information

S1 Video Video Consent for PR-COIN. This consent video was used for the video consent format for this study.
(MP4)

S1 Text Written Informed Consent Form for PR-COIN. This consent form was used for the written informed consent format for this study.
(PDF)

S2 Text Demographic Survey. (TIF)

S3 Text Comprehension Questionnaire. (TIF)

S4 Text Satisfaction Questionnaire. (TIF)

S5 Text Preference Questionnaire. (TIF)

S6 Text Validation Questionnaires. (PDF)
