# Supplementary material for: Video consent is preferred over written informed consent in pediatric rheumatology research
Source: PLOS Digit Health. 2025 Nov 3;4(11):e0001067. doi: 10.1371/journal.pdig.0001067 (PMC12582470; doi:10.1371/journal.pdig.0001067)
Supplement: S1 Text — This consent form was used for the written informed consent format for this study. (PDF) [file pdig.0001067.s002.pdf]

SickKids
THE HOSPITAL FOR
SICK CHILDREN

Patient Research Consent Form

Study Title:
The Pediatric Rheumatology Care and Outcomes Improvement Network Registry (PR-COIN)

Principal Investigator (Study Doctor)
[redacted] MDCM, FRCPC, Division of Rheumatology [redacted]

Co-Investigators:
[redacted], RN, BScN, Division of Rheumatology [redacted]
[redacted], MSc, BScPT, Division of Rheumatology
[redacted], MSc, Practitioner, Division of Rheumatology
[redacted], MD, MSc, FRCPC, Division of Rheumatology
[redacted], BScN, RN, Division of Rheumatology
[redacted] RN, MN, Division of Rheumatology
[redacted], MD, FRCPC, Division of Rheumatology
[redacted], MD, FRCPC, MS, ScD, Division of Rheumatology
[redacted], MD, MS, FRCPC, Division of Rheumatology
[redacted], RN, BScN, Division of Rheumatology
[redacted], MBBCh, FRCPC, Division of Rheumatology
[redacted], MD, FRCPC, Division of Rheumatology
[redacted], MD, FRCPC, Division of Rheumatology
[redacted], MD, FRCPC, Division of Rheumatology
[redacted], MD, PhD, FRCPC, Division of Rheumatology

Study Coordinators/Research Contact:
[redacted], PhD, Division of Rheumatology [redacted]
[redacted], MSc, Division of Rheumatology
[redacted], MSc, Division of Rheumatology

Study Sponsor:
The sponsors/funders of this research are The Arthritis Foundation, The Hinchman Family Fund, The Pediatric Center for Education and Research in Therapeutics at the Cincinnati Children's Medical Center and The Agency for Healthcare Research and Quality.

Conflict of Interest:
There are no conflicts of interest to declare related to this study.

Introduction
We would like to invite you to take part in our research study and quality improvement project. This consent describes the research study and quality improvement project and what it means to participate. Before deciding to take part, please take as much time as you need to ask any questions that you have. You are encouraged to discuss with family, friends, your personal physician or other

health professional, or any members of your community that you trust if that is helpful to you. Participation in any research study is voluntary (you do not have to participate if you don't want to).

Why am I being asked to participate?

You are being invited to participate in this research study and quality improvement project because you have a diagnosis of juvenile idiopathic arthritis (JIA).

Why is this study being done?

We are doing this research project and quality improvement project to try to improve the quality of care that we provide to patients with juvenile idiopathic arthritis (JIA) and their families. Routine information that is collected during clinic visits will be sent electronically (in a secure fashion) to form a registry database (collection of information). The registry will collect and store information on patients with JIA from at least 15 other centres. Researchers will then study the information by comparing how care is delivered and the outcomes of patients at the different centres. By comparing how care is given at each centre and patient outcomes, we can learn how to give better care to our patients and families.

How many participants will be in this study?

As this is a registry, there is no firm number of participants. This registry is currently recruiting patients from Canada and the United States but plans to expand to other countries. We estimate that about 30 new patients will join this study/project at SickKids each year.

How long will the study take?

This registry will be open as long as there is sufficient funding to maintain the registry. There will be ongoing analysis of the information collected in the registry so results may be generated at any time.

What will happen if you join this study?

- You will be asked to sign this consent form. You will be given a copy of this signed consent form to keep and a copy will be placed in your hospital health record.
- Your health record will be accessed and reviewed for your medical information such as lab tests and procedures, clinic notes, medications, treatments, diagnostic tests, physical exams and assessments collected during your clinic visits. We will also collect information on how you manage your condition. This information will be entered into the registry database which will be held in Cincinnati, Ohio, USA. This information will be sent electronically over the internet in a protected way. No additional procedures or testing are involved in this study.
- You will not be identified by name. Every participant will receive a special study identification number. We will collect some of your personal health information such as your medical record number (this information will be retained at SickKids), birth date (only month and year), gender, and race. You will be asked to provide the contact information for other persons who know how to contact you (this information will be retained at SickKids). We will only contact these individuals if we are unable to reach you in the future. We will identify ourselves as being from The Hospital for Sick Children (SickKids) and not reveal any information about your medical condition to these individuals.
- We hope that all children with a diagnosis of JIA at SickKids will join this registry.

This registry does not involve any treatment or intervention for any symptoms. It does not involve extra clinic/hospital visits.

What are the risks, harms or discomforts of the study?
There are no physical risks associated with this registry. There is a potential risk of loss of confidentiality. We will, however, make every effort to protect your confidential information to minimize this risk. You may stop your participation in this registry at any time.

Are there benefits from being in the study?
There are no direct benefits from being in the registry. Research conducted on this information may help us figure out better ways to care for patients with JIA. This might result in better outcomes for you and for other patients with JIA in the future. We will let you know when we learn new things.

Can I choose to leave the study?
You can change your mind at any time during the research study. You do not need to give a reason to withdraw from the study. Withdrawal from the study will not have any effect on the care you or your family receives at SickKids. If you decide to leave the study, you can contact the Principal Investigator or a member of the study team to let them know.
Information that was recorded before you withdrew will be used by the researchers for the purposes of the study, but no information will be collected and sent after you withdraw your permission.

How will your privacy be protected?
We will respect your privacy. PR-COIN is also committed to respecting your privacy. No information about you will be given to anyone or be published without your permission, unless the law requires us to do this.

The SickKids study staff (study investigators, coordinators, nurses and delegates) will collect personal health information about you. This includes things learned from the study procedures described in this consent form and/or information from your medical records. They will only collect the information they need for the study.

All personal health information or personal information collected about you will be "de-identified" by replacing your identifiable information (i.e., name) with a "study number". The SickKids study staff are in control of the study code key, which is needed to connect your personal health information/personal information to you. The link between the study number and your identity will be safeguarded by the SickKids study staff and will not be available to PR-COIN. SickKids guidelines include the following:

- All information that identifies you, both paper copy and electronic information, will be kept confidential and stored and locked in a secure place that only the study staff will be able to access.
- Electronic files will be stored securely on hospital or institutional networks or securely on any portable electronic devices.
- No information identifying you will be allowed off site in any form without your consent. Examples include your hospital or clinic charts, copies of any part of your charts, or notes made from your charts.

The study staff and the others listed above will keep the information they see or receive about you confidential, to the extent permitted by applicable laws. Even though the risk of identifying you from the study data is very small, it can never be completely eliminated.

Access to your personal health information will take place under the supervision of the Study Doctor. You have the right to access, review and request changes to your personal health information.

The following people may come to the hospital to look at your personal health information to check that the information collected for the study is correct and to make sure the study followed the required laws and guidelines:

- PR-COIN; and
- Representatives of the SickKids Research Ethics Board and/or Research Quality and Risk Management team

The study staff will keep any personal health information about you in a secure and confidential location for 7 years after the last publication and then destroy it according to SickKids policy.

When the results of this study are published, your identity will not be disclosed. You have the right to be informed of the results of this study once the entire study is complete.

Any information sent outside of Canadian borders may increase the risk of disclosure of information because the laws in those countries dealing with protection of information may not be as strict as in Canada. However, all study data that are transferred outside of Canada will be coded (this means it will not contain your personal identifying information such as your name, address, medical health number or contact information). Any information will be transferred in compliance with all relevant Canadian privacy laws. By signing this consent form, you are agreeing to the disclosure of your coded information to organizations located outside of Canada.

Will information about this study be available online?
A description of the PR-COIN Network, its initiatives and results are available at any time at https://pr-coin.org. This website will not include information that can identify you.

Will it cost you anything to be in this study?
Participation in this study will not involve any additional costs to you or your private health care insurance.

Will I be paid and/or reimbursed if I join this study?
You will not be compensated for joining this study.

What if I am injured during/in this study?
If you suffer an injury from participation in this study, medical care will be provided to you in the same manner as you would ordinarily obtain any other medical treatment. In no way does signing this consent form waive your legal rights nor release the study doctor(s), sponsors or involved institutions from their legal and professional responsibilities.

How will I be informed about new information?
We may learn new information during the study that you may need to know. We can also learn about things that might make you want to stop participating in the study. If so, you will be notified about any new information in a timely manner. You may also be asked to sign a new consent form discussing these new findings if you decide to continue in the research study.

What are your rights when participating in a research study?
You have the right to receive all information that could help you make a decision about participating in this study. You also have the right, throughout the study, to ask questions about this study and to have them answered to your satisfaction, before you make any decisions.

Will I receive study results?
You have the right to be informed of the results of this registry.
The results of this study will be available on the PR-COIN website as well as information in the SickKids rheumatology clinic.

Your rights to privacy are legally protected by federal and provincial laws that require safeguards to ensure that your privacy is respected.

By signing this form you do not give up any of your legal rights against the study doctor, PR-COIN or involved institutions for compensation, nor does this form relieve the study doctor, PR-COIN or their agents of their legal and professional responsibilities.

You will be given a copy of this signed and dated consent form prior to participating in this study.

Who can I call if I have questions about the study?
If you have any questions during the participation of this research study you can contact [redacted] at [redacted] or any of the study team members listed on the first page.

Research Ethics Board Contact information
The study protocol and consent form have been reviewed by the SickKids Research Ethics Board (REB).

If you have any questions regarding your rights as a research participant, you may contact the Office of the Research Ethics Board at [redacted] during business hours.

Consent to Participate in a Research Study

Study Title: The Pediatric Rheumatology - Care and Outcomes Improvement Network Registry (PR-COIN)

By signing this research consent form, I understand and confirm that:

1. The study has been explained to me and all of my questions have been answered.
2. I have the right not to take part in the study.
3. I can stop participating or withdraw from the study at any time without affecting the quality of care I or my family receives at SickKids.
4. The possible harms and benefits (if any) of this study have been explained to me.
5. I have been told that my medical records will be kept private except as described to me.
6. I know that no identifying information about me will be given to anyone or be published without first asking permission, unless required by law.
7. I have been told that I have not waived my legal rights nor released the investigators, sponsors, or involved institutions from their legal and professional responsibilities.
8. I have been given sufficient time to read and think about the information in this consent form.
9. I know that I may ask now, or in the future, any questions I have about the study.
10. I have been told that I will be given a signed and dated copy of this consent form.

I consent to participate in this study.

______________________	______________________	______________________
**Printed Name of Participant**	**Participant's signature**	**Date**

______________________	______________________	______________________
Printed Name of person who obtained consent	***Signature of Person who obtained consent***	***Date***

The person signing below acted as an interpreter, and attests that the study as set out in the consent form was accurately sight translated and/or interpreted, and that interpretation was provided on questions, responses and additional discussion arising from this process.

______________________	______________________	______________________
Printed Witness's name (if the parent/legal guardian does not read English)	*Witness's signature*	*Date*

____

Language
