# Supplementary figures and images for: Video consent is preferred over written informed consent in pediatric rheumatology research
Source: PLOS Digit Health. 2025 Nov 3;4(11):e0001067. doi: 10.1371/journal.pdig.0001067 (PMC12582470; doi:10.1371/journal.pdig.0001067)

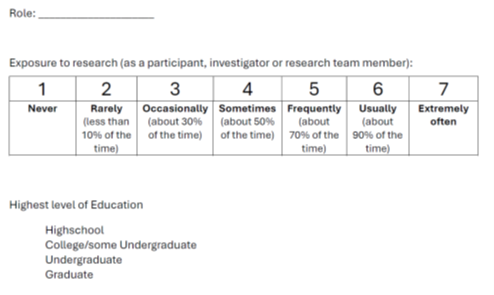

Supplement: S2 Text — (TIF) [file pdig.0001067.s003.tif]

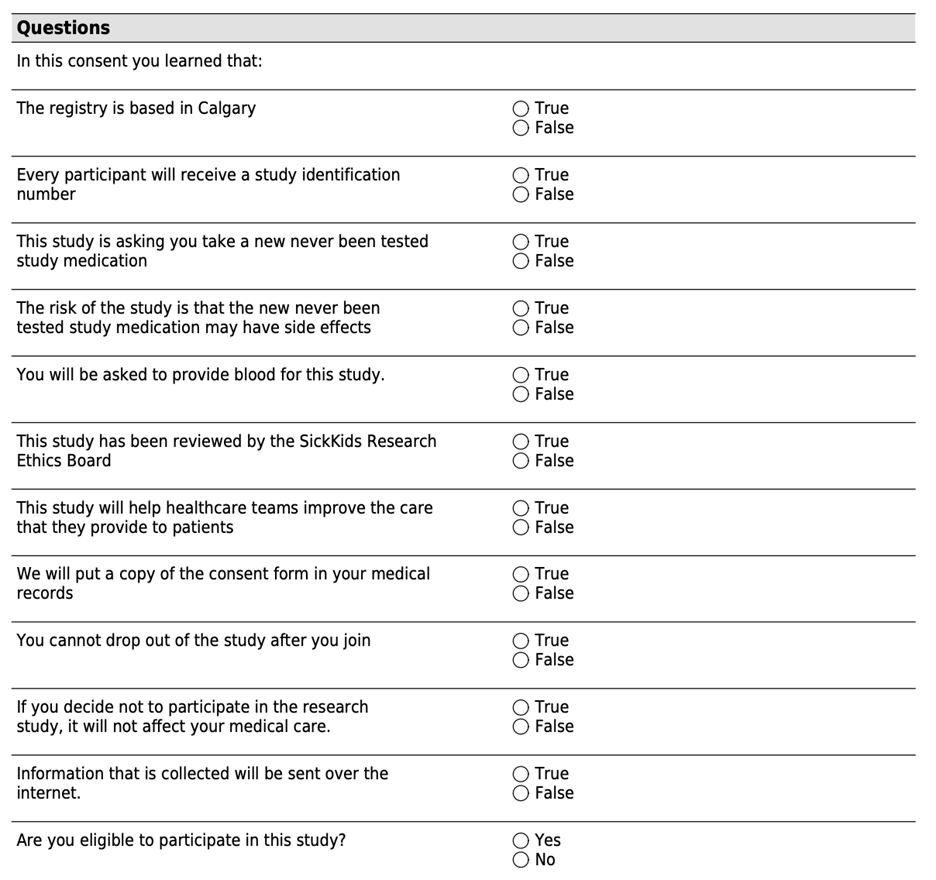

Supplement: S3 Text — (TIF) [file pdig.0001067.s004.tif]

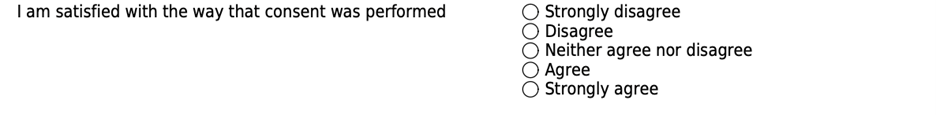

Supplement: S4 Text — (TIF) [file pdig.0001067.s005.tif]

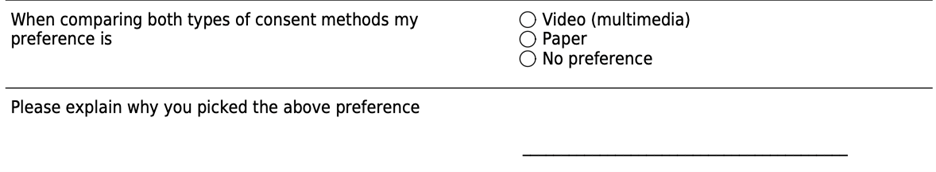

Supplement: S5 Text — (TIF) [file pdig.0001067.s006.tif]
