# Supplementary material for: Video consent is preferred over written informed consent in pediatric rheumatology research
Source: PLOS Digit Health. 2025 Nov 3;4(11):e0001067. doi: 10.1371/journal.pdig.0001067 (PMC12582470; doi:10.1371/journal.pdig.0001067)
Supplement: S6 Text — (PDF) [file pdig.0001067.s007.pdf]

Do you think this questionnaire accurately measures a person's comprehension of the PR-COIN research consent form?

Strongly Disagree	Disagree	Somewhat Disagree	Neither Agree nor Disagree	Somewhat Agree	Agree	Strongly Agree

Do you think this questionnaire accurately measures a person's satisfaction after an informed consent experience?

Strongly Disagree	Disagree	Somewhat Disagree	Neither Agree nor Disagree	Somewhat Agree	Agree	Strongly Agree

Do you think this questionnaire accurately measures which consent process a person prefers?

Strongly Disagree	Disagree	Somewhat Disagree	Neither Agree nor Disagree	Somewhat Agree	Agree	Strongly Agree
